# Robust Gaussian Process Regression Method for Efficient Tunneling Pathway Optimization: Application to Surface Processes

**DOI:** 10.1021/acs.jctc.4c00158

**Published:** 2024-05-06

**Authors:** Wei Fang, Yu-Cheng Zhu, Yihan Cheng, Yi-Ping Hao, Jeremy O. Richardson

**Affiliations:** † Department of Chemistry, Shanghai Key Laboratory of Molecular Catalysis and Innovative Materials, Fudan University, Shanghai 200438, P. R. China; ‡ Laboratory of Physical Chemistry, ETH Zürich, Zürich 8093, Switzerland; § State Key Laboratory of Molecular Reaction Dynamics and Center for Theoretical Computational Chemistry, Dalian Institute of Chemical Physics, Chinese Academy of Sciences, Dalian 116023, P. R. China; ∥ State Key Laboratory for Artificial Microstructure and Mesoscopic Physics, Frontier Science Center for Nano-optoelectronics and School of Physics, Peking University, Beijing 100871, China; ⊥ Department of Chemistry and Applied Biosciences, ETH Zürich, Zürich 8093, Switzerland

## Abstract

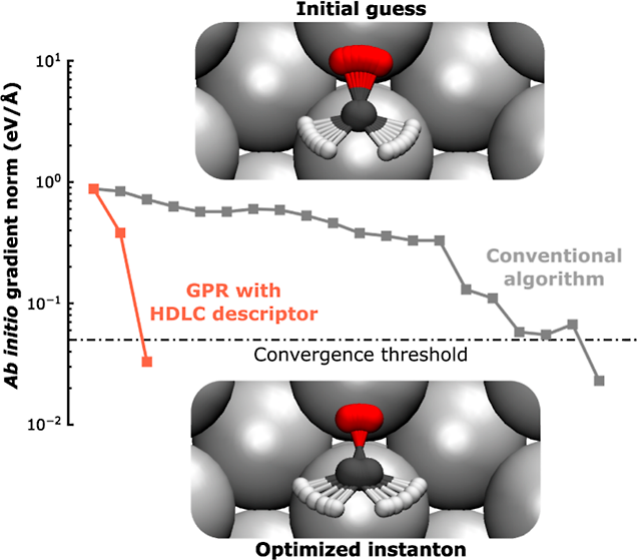

Simulation of surface processes is a key part of computational chemistry that offers atomic-scale insights into mechanisms of heterogeneous catalysis, diffusion dynamics, and quantum tunneling phenomena. The most common theoretical approaches involve optimization of reaction pathways, including semiclassical tunneling pathways (called instantons). The computational effort can be demanding, especially for instanton optimizations with an ab initio electronic structure. Recently, machine learning has been applied to accelerate reaction-pathway optimization, showing great potential for a wide range of applications. However, previous methods still suffer from numerical and efficiency issues and were not designed for condensed-phase reactions. We propose an improved framework based on Gaussian process regression for general transformed coordinates, which has improved efficiency and numerical stability, and we propose a descriptor that combines internal and Cartesian coordinates suitable for modeling surface processes. We demonstrate with 11 instanton optimizations in three representative systems that the improved approach makes ab initio instanton optimization significantly cheaper, such that it becomes not much more expensive than a classical transition-state theory rate calculation.

## 1 Introduction

Surface processes and reactions are at the core of many important phenomena, such as heterogeneous catalysis, ice nucleation, and corrosion, to name just a few. Computer simulations have been essential for understanding surface structure, reactions and dynamics on surfaces, as well as quantum tunneling phenomena in these systems, achieving tremendous success.^1−3^ Locating reaction pathways (typically minimum-energy pathways) is one of the most crucial parts of modern computational research, offering insights into the mechanism of surface processes and reactions.^4^

As the demand for higher accuracy modeling increases, incorporation of nuclear quantum effects, in particular, quantum tunneling, in simulations is becoming the new standard. Ring-polymer instanton theory is a robust method for rigorously including tunneling effects into the simulation of reaction rates and mechanisms^5−10^ as well as for computing tunneling splittings,^10,11^ with a good balance between accuracy and efficiency. It employs a semiclassical approximation to define a dominant tunneling pathway called an instanton, which can be located via a first-order saddle-point optimization on the ring-polymer potential-energy surface. It is important to note that this pathway is not, in general, equivalent to the minimum-energy pathway. The instanton rate only requires local properties (i.e., potentials, gradients, and Hessians) along the tunneling pathway (instanton), due to the semiclassical approximation. Thus, ring-polymer instanton theory can be viewed as a quantum-mechanical extension of the well-known classical transition state theory (TST). Using instanton theory, recent studies have unveiled several interesting phenomena on different surfaces related to quantum tunneling,^12−18^ demonstrating the importance of modeling quantum tunneling in the simulation of surface processes. Although instanton theory is much more efficient than a full quantum calculation, it is still more demanding than classical TST, especially when combined with ab initio electronic-structure calculations, which has so far impeded the wide application of rigorous tunneling calculations. Therefore, reducing the cost of the optimization of tunneling pathways (instantons) is important to the advance of this field.

Earlier works on improving optimization schemes were mostly dedicated to finding better coordinate systems in which to perform the optimization,^19−24^ or on improving the approximate Hessian of the system to accelerate convergence.^25,26^ Yet, the past decade has witnessed a remarkable emergence of machine-learning techniques in computational chemistry, unveiling a vast realm of new possibilities. In recent years, machine learning methods have been applied to geometry optimizations,^27−33^ challenging the conventional algorithms that have stood for decades. These methods use machine learning to fit the local potential-energy surface (PES) around a local minimum geometry or the dominant reaction/tunneling pathway and perform optimizations on the fitted PES. By iterating this procedure, they can be converged to give the same pathway as for the true PES at a fraction of the cost. Note that this application is very different from fitting a global PES with machine learning;^34−36^ it requires very high accuracy learning in a small local region of the phase space, with a very small training data set in the region. For instanton optimization, in addition to the learning and prediction of energies and gradients that are commonly done with machine learning, Hessian training and prediction is also practically crucial.^31^ Methods based on both neural networks (NN) and Gaussian process regression (GPR) have been explored. The predictive performance of GPR has been shown to be very competitive,^31,37^ especially with limited training data;^38^ therefore, it is preferred for this application.

Despite the great success of GPR-assisted optimization methods demonstrated in previous work, there are still several issues that limit their feasibility, especially for application to surface systems. A major issue is that since they were mostly designed for reactions in the gas phase (which requires translational and rotational invariance), the descriptors used are not applicable to surface systems. In addition, there are a few practical issues. First, the previous GPR method suffers from numerical stability problems for planar and linear molecules when using bond-based internal coordinate descriptors. Second, memory and efficiency issues caused by Hessian training or the use of long descriptors (such as redundant internals) can plague the performance in relatively large systems. Therefore, further methodological improvements of GPR optimization schemes are urgently needed.

In this work, we develop an improved GPR method for geometry optimization that (i) is suitable for modeling surface reactions and processes; (ii) can be applied to instanton optimization (i.e., it includes Hessian training); and (iii) addresses the previous practical issues. We test the performance of our method on three systems each covering a different type of surface process: (a) H_2_O dissociation on Cu(111), a representative surface catalysis reaction; (b) CH_2_O rotation on Ag(110), a dynamical process on the surface that can be observed in STM experiments; (c) double proton transfer (DPT) in the formic acid dimer (FAD) on NaCl(001), a reaction between adsorbates on the surface. Good performance is observed for GPR-assisted instanton optimization in these test systems, showing fast convergence of ab initio gradients with just a few iterations. Our approach also alleviates some of the previous practical issues, making GPR optimizations more robust. In particular, we show that “selective Hessian training” (available within the method introduced in this work) provides a computational advantage for the application to larger systems. The improved approach retains the accuracy of the original instanton method as the results are formally identical once the algorithm is converged, while requires far less computational effort than an instanton optimization carried out with conventional methods.

## 2 Theory

### 2.1 Ring-Polymer Instanton Theory

Quantum mechanical reaction rates can be rigorously defined using the flux correlation function.^39^ Instanton theory can be derived by taking semiclassical approximations to the flux correlation function.^10^ Here, we skip over the derivation and present the final instanton rate expression
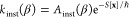
1in which  is the inverse temperature, **x** is the minimum-action tunneling pathway with imaginary time β*ℏ* called an instanton, *S* is its Euclidean action, and *A*_inst_ is a prefactor term that mainly characterizes the fluctuations around the instanton. The instanton can be represented by a *N* bead ring polymer, **x** = {x_1_, ..., x_*N*_}, and its Euclidean action is given by the ring-polymer potential
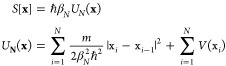
2where β_*N*_ ≡ β/*N* and *x*_0_ ≡ *x*_*N*_. The ring-polymer instanton **x** can be calculated via first-order saddle-point optimization of *U*_*N*_(**x**). Finally, to compute the rate, one also needs to perform Hessian calculations on the optimized instanton **x**, which is needed for computing *A*_inst_.

In addition to computing reaction rates, instanton theory can also be applied to efficiently compute tunneling splittings for molecules with permutationally degenerate wells.^10,11^ The procedure is very similar to that of instanton rate calculations, which also requires optimization of minimum-action tunneling pathways (instantons). Therefore, the development of efficient methods for optimizing tunneling pathways is crucial and holds tremendous potential for various applications.

### 2.2 Gaussian Process Regression

GPR is a machine-learning algorithm which can be used to efficiently generate complex hypersurfaces with limited data.^40^ In recent years, this method has been applied for constructing global PESs from data generated from ab initio calculations,^34,37^ and for geometry and reaction pathway optimizations.^30−33^ For geometry, reaction pathway, or tunneling pathway optimizations, one constructs a high accuracy local PES around the minimum, nudged elastic band, or instanton pathway using GPR from a small training set generated from ab initio calculations performed on the initial guess. The optimization is performed via an iterative process that mainly includes two steps: performing optimization on the GPR PES and adding ab initio data to the GPR PES, which we describe in detail in Section 4.2. Here, we give a brief introduction to GPR.

Given a training set of *M* geometries {**x**_*m*_} and potential energies {*V*(**x**_*m*_)} ({*V*_*m*_} for short), the GPR prediction of the potential energy for a new geometry x* is
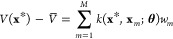
3where *V̅* is the average potential of the training set, *k* is the kernel function, **θ** is a vector of hyperparameters, and  are the weights. There are many possible choices for the kernel function;^40^ in this work, we use the Gaussian kernel *k*(**x**_*i*_, **x**_*j*_) = θ_1_ exp(−θ_2_∥**x**_*i*_ – **x**_*j*_∥^2^). The weights are determined by solving a set of linear equations , where **K** is the covariance matrix with element *k*(**x**_*i*_, **x**_*j*_) in the *i*-th row and *j*-th column, **I**_*M*_ is the identity matrix of rank *M*, and σ is the noise hyperparameter.

### 2.3 Gradient and Hessian Learning with GPR

For geometry optimization, learning and predicting gradients are important, while for instanton optimizations, Hessian learning and prediction also become necessary in practice. This can be achieved with an extension^30^ to eq 3
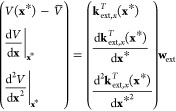
4

For the sake of clarity and convenience, we will define a set of conventions here.

- **bold lower case**: column vector.
- **bold upper case**: matrix.
: column vector of length *f*_*x*_ (*f* denotes the length of the vector noted in the subscript).
: column vector of length *f*_*Hx*_ = *f*_*x*_(*f*_*x*_ + 1)/2. This is the upper triangle of the Hessian matrix, flattened into an array in the row-major ordering.
: matrix with *f*_*x*_ rows and *f*_*k*_ columns.
: matrix with *f*_*Hx*_ rows and *f*_*k*_ columns.

Also, we order the training data such that the first *M*_*g*_ entries have gradient data, and the first *M*_*H*_ ≤ *M*_*g*_ entries additionally have Hessian data. Within this notation the terms in eq 4 can be written as
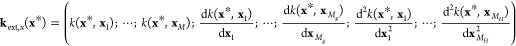
5(the subscript *x* denotes that the kernel derivatives are taken with respect to Cartesian coordinates). **w**_ext_ are the extended weights, obtained by solving the following set of linear equations
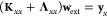
6in which
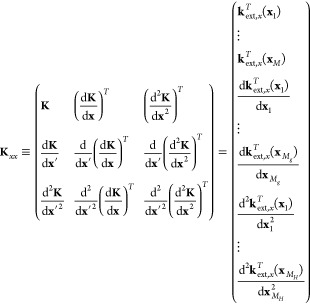
7is a symmetric extended covariance matrix
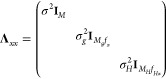
8is the extended noise matrix, and
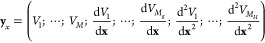
9is the training data.

### 2.4 GPR with General Descriptors

Constructing GPR potentials using the Cartesian coordinate is the simplest and most straightforward way. However, it has some drawbacks; for example, when describing gas-phase molecules, the Cartesian coordinates are not translationally or rotationally invariant. Moreover, it is not a natural way to describe bonding in molecules. Since the descriptors are arguably the most important part of building a good machine-learning potential,^35,41^ it is desirable to design a GPR method for general descriptors **q**. This idea has been probed for certain coordinate systems, such as the redundant and delocalized internal coordinates.^19^ The GPR method designed in previous works^31,33^ functions in the following manner: all observables (gradients and Hessians) are transformed from Cartesian coordinates into internal coordinates in the training process. In the prediction process, it first predicts gradients and Hessians in internal coordinates, then transforms them back into Cartesian gradients and Hessians, and finally rotates them to match the orientation of the input geometry. This approach has some drawbacks; e.g., it breaks down for planar and linear molecules if bond-based internal coordinate descriptors are used. Also, the back and forth transformations from **x** to **q** for the forces and Hessians are nontrivial and may encounter numerical issues. A second approach, inspired by the work of Bartók et al.,^37^ has been explored recently,^32^ which avoids the transformation of the forces and instead applies the transformation to the covariance matrix. However, the second approach has only been developed for gradient training, which is applicable to geometry optimization but is inadequate for instanton optimizations. Here, we extend the second approach to Hessian training and prediction, and furthermore, we show that the two approaches can in fact be unified (or reformulated) into one framework.

We define a general (nonlinear) descriptor **q** as
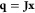
10where **J** is the Jacobian matrix
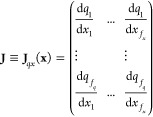
11

We use the Gaussian kernel in the **q** coordinate representation
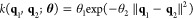
12

When the kernel is defined in the transformed coordinates **q**, the extended covariance matrix **K**_*xx*_ can be constructed by applying a transformation matrix **L**, where **K**_*xx*_ = **LK**_*qq*_**L**^*T*^. **K**_*qq*_ is defined similarly as **K**_*xx*_, but with the kernel derivatives taken with respect to **q** instead of **x**, which follows directly from differentiating eq 12. **L** is derived using the chain rule, and it can be formally written as
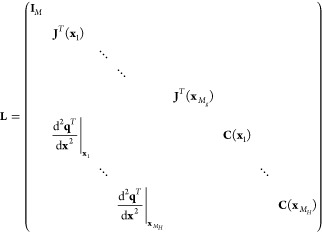
13in which the elements in **C** are given by
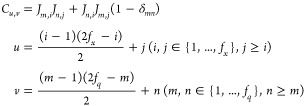
14

Finally, the training process of the transformed GPR proceeds via solving
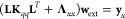
15and computing . The prediction step thus becomes
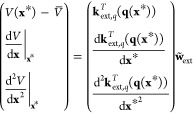
16in which **k**_ext,*q*_ is defined similarly as **k**_ext,*x*_ (eq 5), but with the kernel derivatives taken with respect to **q** instead of **x**. Hyperparameter optimization for the coordinate transformed GPR can be done via maximizing the log marginal likelihood,^40^ which is the same as that for GPR without coordinate transformation.

The coordinate transformed GPR inherits desirable properties of the descriptor **q**. For example, if **q** are the internal coordinates,^19^ then one can see that the energy predictions of the GPR model (eq 16) are translationally and rotationally invariant, and that the predicted Cartesian gradients and Hessians are rotated correctly. Compared to the previous GPR method for instanton optimization,^31^ the GPR framework developed here possesses several desirable features. It does not suffer from numerical instabilities for planar molecules. Equation 16 avoids the back and forth transformation of the gradients and Hessians from Cartesian coordinates to **q** coordinates, reducing complexity and improving the numerical stability. Equation 15 allows training with Hessian data of selected degrees of freedom (selective Hessian training), instead of using the full Hessian, which can be very practical since Hessian training is the most expensive and memory demanding part of GPR. Selective Hessian training can be implemented by simply eliminating rows from **L** (eq 13) that correspond to the elements discarded from the full Hessian. Another feature is that the memory cost and computational cost now scales with the length of **y**_*x*_ instead of **y**_*q*_, i.e.,  and , respectively, which can make the use of longer descriptors more feasible. The scaling of the computational cost is determined by solving the linear equations in eq 15 (in the case *f*_**y**_*q*__ > *f*_**y**_*x*__^3/2^, the scaling becomes , which is the cost of constructing **K**_*qq*_), while the cost of coordinate transformation should not become the bottleneck since **L** is sparse.

Furthermore, we show that the previous approach^31^ can be unified into the GPR framework presented in this work. Instead of solving eq 15 in the training process, one can alternatively solve the following linear equations
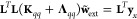
17

The prediction step is the same as that in eq 16. This is equivalent to the previously proposed GPR method,^31^ while also avoiding the transformation of the physical quantities (i.e., forces and Hessians). However, this formulation does not resolve the numerical instability issue of the previous GPR method for planar molecules. Comparing eqs 15 and 17, the main difference lies in the noise matrix **Λ**. We infer that adding the noise matrix to **K**_*xx*_ instead of **K**_*qq*_ is key to resolving the numerical instability issue for planar molecules, while that where the coordinate transformation is applied might not be so crucial. We believe that this unification advances our understanding of coordinate transformation in GPR, and creates possibilities for future improvements, e.g., via modifying eqs 15 or 17.

### 2.5 Descriptors for GPR Learning of Surface Systems

We consider systems with an adsorbed molecule (or a cluster) on a given surface. Using Cartesian coordinates as the descriptor is a viable option; however, it is not an intrinsic descriptor for describing covalent bonds in the adsorbate. Meanwhile, internal coordinates can describe the adsorbate well but are not suitable as this type of system is neither translationally nor rotationally invariant. To describe the translation and the rotation of the adsorbate, one requires at least 6 variables, i.e., the coordinates of the centroid (**x**_c_) and 3 rotation angles. Intuitively, one would consider using the Euler angles; however, we found that this can be problematic. One can imagine a simple case, a small rotation α about the *y* axis, the Euler angles (using the standard “ZXZ” convention) for this rotation is (π/2, α, – π/2). Under the metric of distance in the kernel (eq 12), the Euler angles would suggest that the rotated structure is far from the initial structure, which is unfaithful. This means that, at the very least, the kernel needs to be redefined to resolve this issue. Moreover, since the adsorbate molecule is not rigid and may even dissociate in surface processes that we intend to model, Euler angles might not even be well-defined and might be sensitive to the choice of the reference structure.

Instead of trying to come up with a good descriptor for rotations, we worked around this issue. We propose an idea that combines the internal coordinates and Cartesian coordinates in order to “gain the best of both worlds”. Here, we use a specific definition of internal coordinates (which are the pairwise atomic distances) instead of the general definition (which includes angles and dihedrals) such that the internal coordinates have the same units as Cartesian coordinates. First, we divide the system into two parts: the adsorbates (with a total of *N*_a_ atoms) and the flexible substrate atoms (*N*_s_ atoms). We also make sure that all of the atoms are not wrapped by periodic boundary conditions. Combining the Cartesian coordinates and the internals of the adsorbate gives the following descriptor
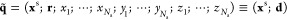
18where **x**^s^ are the Cartesian coordinates of all the flexible substrate atoms, **d** are the coordinates representing the adsorbate and its relation with the substrate, and
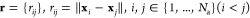
19**q̃** is obviously redundant, and it is necessary to trim out the redundancy. We use a method similar to the method for constructing the delocalized internals from redundant internals.^19^ One can perform singular value decomposition on the Jacobian matrix from **x** to **d** (**J**_*dx*_). Since **J**_*dx*_ is **x** dependent, previous works selected a reference geometry from the training set. Alternatively, we can average **J**_*dx*_ over all of the geometries in the training set
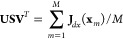
20

The delocalization matrix **B**_*qd*_ is constructed by taking the row vectors in **U**^*T*^ that correspond to the nonzero singular values in **S**. Therefore, the nonredundant descriptor is given by
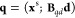
21

We refer to **q** as a mixed internals and Cartesian (MIC) descriptor. **q** reduces to Cartesian coordinates when the adsorbate is a single atom. Our GPR PES for surface systems is built using eqs 21, 15, and 16.

It is useful to discuss some of the other descriptor options that we have considered and the reason why they were not chosen. First, we note that internal coordinates often use the 1/*r*_*ij*_ instead of *r*_*ij*_. It is also feasible to construct **q** (eq 21) using 1/*r*_*ij*_; however, mixing 1/*r*_*ij*_ with Cartesian coordinates will result in inconsistent units in the descriptor. Another idea is to select reference points on (or near) the surface and characterize the translations and rotations using the distances of the adsorbate atoms from the reference points. We found that the performance of this approach is sensitive to the choice of the reference points, and arbitrary reference usually leads to mediocre performance; therefore, we do not recommend it despite it being feasible.

We note that **q** is similar to the hybrid delocalized internal coordinates (HDLC) proposed in the 2000s for reducing the computational scaling of constructing delocalized internal coordinates (DLC) for large molecules.^22^ HDLC uses a divide-and-conquer approach that breaks a large molecule into fragments to achieve linear scaling with respect to the molecule size. For each fragment, the internal coordinates are supplemented with Cartesian coordinates and then delocalized to generate HDLC. The step in HDLC that mixes internal coordinates and Cartesian coordinates is indeed the same as what we do to describe the adsorbent molecules on a surface. Since MIC certainly belongs to the HDLC family of descriptors, we use the name HDLC (in a broad sense) instead of MIC in the manuscript.

The HDLC descriptor is not limited to describing surface systems; for example, it can also be directly applied to describe reactions/processes in solids. In this case, **x**^s^ would represent flexible atoms in the surroundings of the core reaction region, instead of the substrate atoms. Further, HDLC-like descriptors are not limited to mixing internal coordinates and Cartesian coordinates. One can view that the Cartesian coordinates in **d** (eq 18) alternatively as coordinates describing the connection between the adsorbate and the substrate. Thus, one can replace them with other coordinates designed to describe the connection between the core region and environment and construct **q** (eq 21) from that. These extensions can make HDLC-like descriptors useful for a wide range of systems, such as general systems that can be divided into a core region plus an environment.

## 3 Computational Setup

Electronic structures for the three test systems, namely, H_2_O on Cu(111), CH_2_O on Ag(110), and FAD on NaCl(001), are described with density-functional theory (DFT). Our DFT calculations were carried out using the Vienna ab initio simulation package (VASP).^42,43^ The optB86b-vdW exchange–correlation functional,^44,45^ which accounts for van der Waals interactions, was used. A plane-wave cut off of 400 eV was used (550 eV was used for the FAD system). The Cu(111) substrate was modeled using a four-layer slab in a 3 × 3 supercell. The Ag(110) substrate was modeled using a 4-layer slab in a 3 × 4 supercell. The NaCl(001) substrate was modeled using 2-layer slab in a 2 × 2 supercell. We used a 3 × 3 × 1 *k*-point mesh for all the systems. A vacuum of at least 12 Å was placed above each slab, and a dipole correction was applied along the *z* axis. The substrates were prepared with the top two layers relaxed (top one layer relaxed for NaCl). The climbing image nudged elastic band (CI-NEB) method^4^ was used to compute the minimum-energy pathways (MEP). During the geometry optimization, including CI-NEB and instanton calculations, four (two) substrate atoms closest to H_2_O (CH_2_O) were also optimized, while the other substrate atoms are kept frozen. For FAD, the substrate was kept frozen. The convergence criterion for geometry optimizations and CI-NEB calculations was to converge the maximum force component to below 0.02 eV·Å^–1^. For our instanton optimizations, the convergence criterion was to converge the total gradient to below 0.05 eV·Å^–1^.

## 4 Results

### 4.1 Analysis of the Descriptors

**Figure 1:**
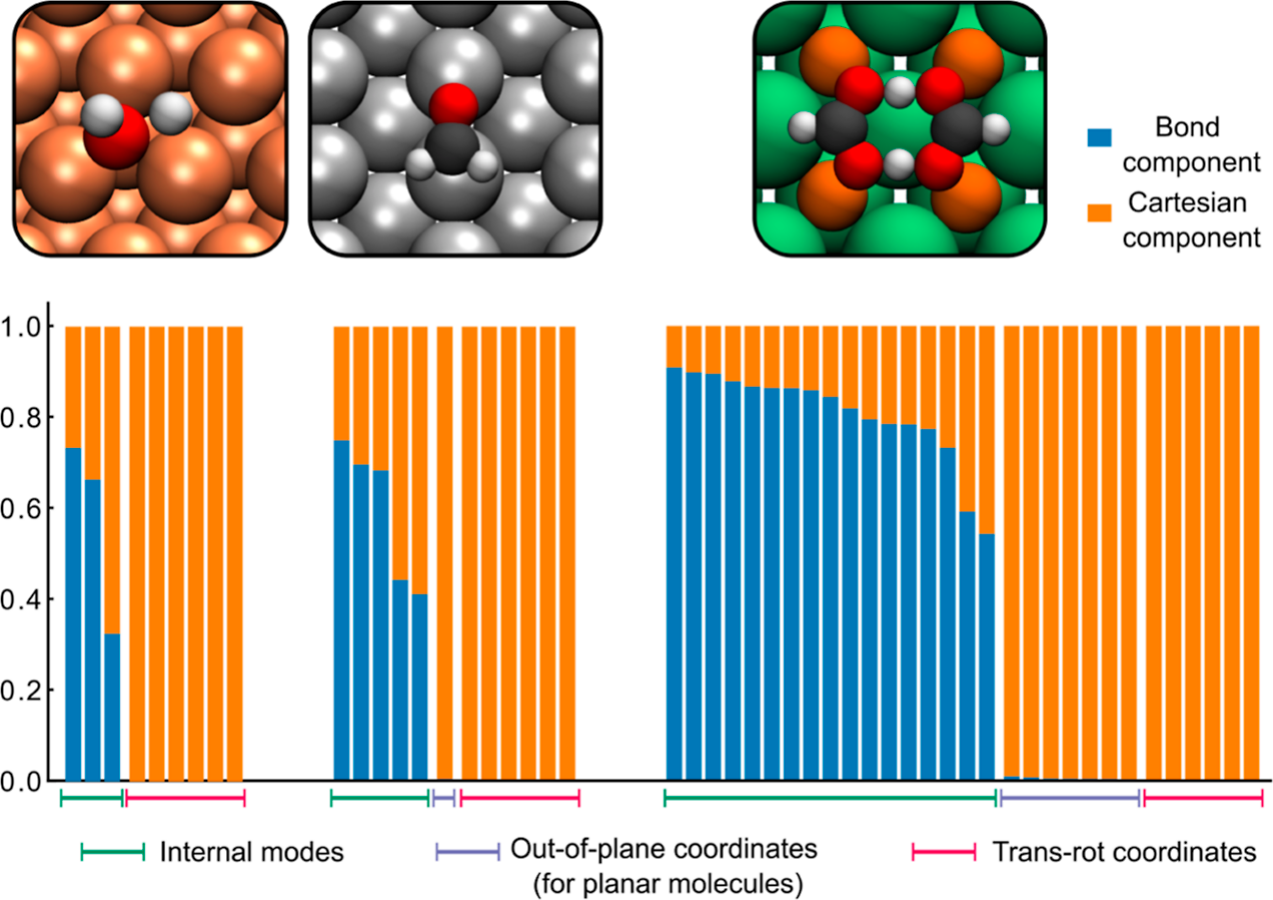
Bond and Cartesian components of HDLC descriptors for the three systems. The transformation matrix (eq 20) is computed using geometries on the CI-NEB path. The bars show the unitary singular vectors (rows in **B**_*qd*_) arranged according to their singular values in descending order. The bond component is the sum of the square of the first *N*_*a*_(*N*_*a*_ – 1)/2 terms in a singular vector, and the Cartesian component is the sum of the square of the remainder terms. The top panels show the classical TS for the three systems.

First, we examine the HDLC descriptors for the three test systems via a decomposition of the adsorbate-related elements in the descriptor (Figure 1). For H_2_O on Cu(111), it has 3 nonredundant internal coordinates and 6 translational and rotational coordinates. In the HDLC descriptor, the first 3 elements (which has the highest 3 singular values) have a significant portion of bond components, meaning that they correspond to the internals of the molecule. The remaining 6 elements are purely linear combinations of Cartesian coordinates, with 3 corresponding to the centroid of the molecule representing translation and the other 3 representing rotations. For CH_2_O on Ag(110), since the molecule is planar, 5 of its 6 internal coordinates are nonredundant. Correspondingly, the first 5 elements of the HDLC descriptor have significant bond components. The sixth element consists of *z* coordinates, representing the out-of-plane mode of the adsorbate. The final 6 elements represent the translation and rotation of the adsorbate. FAD is also a planar adsorbate, so while it has *N*_*a*_(*N*_*a*_ – 1)/2 = 45 redundant internal coordinates, only 2*N*_*a*_ – 3 = 17 are nonredundant. Its HDLC descriptor fully covers all the nonredundant internal coordinates, having 17 elements that mainly consist of bond components. HDLC descriptor also has 7 elements that consist of majorly *z* coordinates, representing out-of-plane modes of the adsorbate. These elements together with the 17 “bond” elements make up the 3*N*_*a*_ – 6 = 24 modes representing the adsorbate, and the remainder 6 elements are the translational and rotational coordinates. The above analysis shows that the HDLC descriptor not only mixed the bond and Cartesian coordinates but also mixed them in an appropriate manner that gives a faithful description of the system. We show later in this article that it is indeed advantageous over Cartesian coordinates for GPR modeling of surface systems.

### 4.2 General Workflow

**Figure 2:**
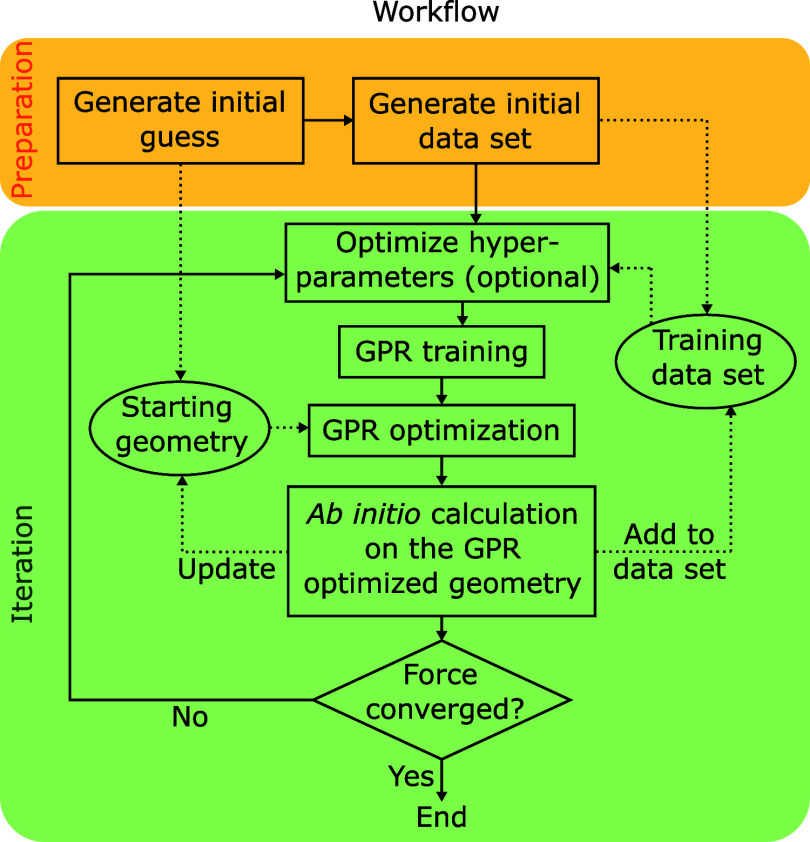
Workflow chart for GPR assisted geometry optimization. The dotted arrows show the flow of data (i.e., geometries and ab initio data).

In this section, we describe the workflow for geometry optimization with GPR. The whole procedure is divided into two parts, “preparation” and “iteration” (Figure 2). For all geometry optimizations, one needs to prepare an initial guess geometry, and with the GPR approach, the initial data set is generated on the initial guess geometry. Then, we optimize the hyperparameters of GPR. This step is optional, as there are methods for obtaining a good set of hyperparameters without optimization, and that it has been shown that the log marginal likelihood is not very sensitive to the hyperparameters as long as they are in a reasonable range.^33^ Since hyperparameter optimization can be computationally inefficient, we recommend to optimize it once for the initial data set and check the performance of GPR on the training data set every time new data is added to determine whether the hyperparameters need to be reoptimized.

With the initial data set and a good set of hyperparameters ready, we can iteratively build the GPR PES and perform optimizations on it. A quasi-Newton optimization algorithm adapted for instanton optimizations is used, which has been shown to be arguably the most stable optimization method for instantons.^46,47^ Optimization proceeds until either the total force on the GPR PES reaches the convergence threshold or an early stop condition is met. Different early stop conditions could be defined for different applications;^30^ in this work, an early stop is triggered if the total force increases for more than three consecutive steps. When an early stop is triggered for the first time, we attempt the GPR optimization a second time from where it stopped. Once the early stop is triggered again, we take the geometry at the step before the consecutive force increase occurred as the final geometry of the optimization step. After optimization on the GPR PES, we compute the true ab initio energy and forces on the final geometry to check whether the forces have reached our convergence criteria. If not, we add the newly computed ab initio data to the training set and repeat the iteration steps (Figure 2). Note that one could also perform a data refinement, which removes some of the data (e.g., data added in early iterations) to improve efficiency.

### 4.3 Instanton Optimization

**Figure 3:**
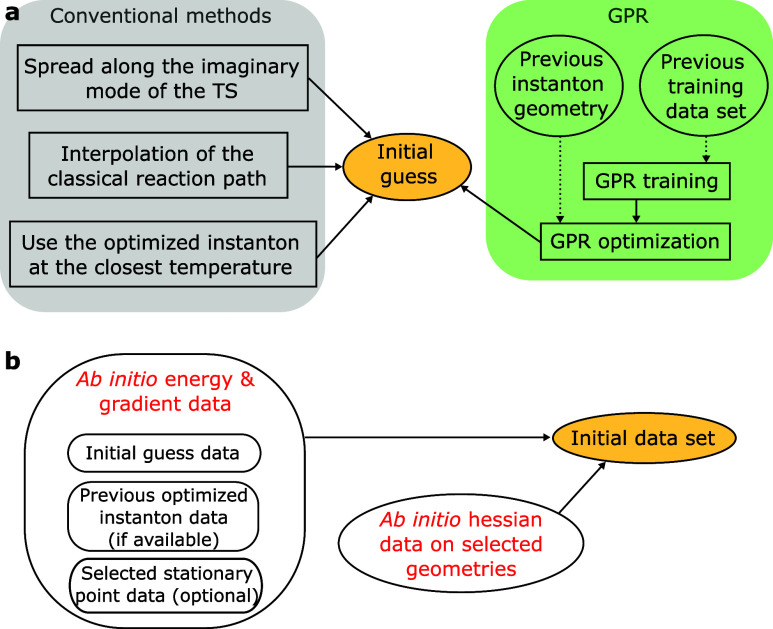
(a) Conventional methods vs the GPR method proposed in this work for generation initial guesses for instanton optimization. (b) Components of the initial data set for instanton optimization used in this work.

For instanton optimization, the methods proposed in this work for generating initial guess geometry and an initial data set are shown in Figure 3. Conventionally, the initial guess for instanton optimization is generated via “spreading” the beads along the imaginary mode of the TS^46^ or via interpolation of CI-NEB images (i.e., the climbing image and a few adjacent images). If an optimized instanton configuration is available at a temperature not too far from the target temperature, then conventionally, it is a good choice to start the instanton optimization from that configuration. Therefore, if the goal is to obtain instantons at different temperatures, one would optimize them in a “sequential cooling” manner,^46^ i.e., start with instanton optimization at the highest target temperature and perform instanton optimizations in a descending order according to temperature. We propose a GPR based approach for generating an initial instanton guess when there is instanton data at another temperature available. We train a GPR PES using the previous instanton data and perform instanton optimizations on the GPR PES at the target temperature. Typically, this GPR optimization could not reach the target force convergence and ended via an early stop with the criteria described in the previous section. We show later in this section that this is a good approach with the HDLC descriptor but not with the Cartesian descriptor.

After obtaining the initial instanton guess, we generated our initial data set for GPR training based on a simple protocol (as described in Figure 3b). The initial data set is composed of three parts: data of the beads of the initial guess, data from a previously optimized instanton at an adjacent temperature (if available), and (optionally) selected stationary point (e.g., reactant, TS, product) data. All data points have ab initio energy and gradient data, while ab initio Hessian data are added for selected geometries. Specifically, the geometries with Hessian data include the first, final, and the highest energy beads from the initial instanton guess, and the first and final beads from the previous instanton (if available). Hessian data of the reactant, TS, and product geometries can also be included, depending on whether they are close to the instanton.

We give the detailed settings for our GPR assisted instanton optimizations in Table 1. The aim of this work is to demonstrate the performance of our method for different types of systems, meanwhile covering the shallow tunneling to deep tunneling regimes. Therefore, three temperatures are chosen for each system (two for FAD) such that at the highest temperature, thermally activated tunneling near the barrier top occurs; at the middle temperature, tunneling through the middle of the barrier occurs; and at the lowest temperature, deep tunneling occurs. At each temperature, we used the minimal number of beads that can reasonably represent the tunneling pathway. In a later section, we show how GPR can be used to extrapolate instantons from a small number of beads to a large number of beads. The sizes of the initial data sets generated with the protocol in Figure 3b are given in Table 1, which are modest and do not vary much for different systems.

**Table 1:** Settings and Initial Data Set Components, i.e., the Number of Energy and Gradient Data Points (*n*_ener,grad_) and the Number of Hessian Data (*n*_hess_), for Each GPR-Assisted Instanton Optimization^a^

system	*T* (K)	*N* beads	*n*_ener,grad_	*n*_hess_
H_2_O–Cu(111)	200	14	16	3
(*T*_c_ ∼ 281 K)	130	30	31	6
	80	50	49	6
CH_2_O–Ag(110)	18	14	14	5
(*T*_c_ ∼ 22 K)	12	30	29	8
	8	50	47	8
FAD–NaCl(001)	150	14	14	5
(*T*_c_ ∼ 223 K)	100	30	29	8

a The crossover temperature to quantum tunneling, estimated by , where ω^⧧^ is the imaginary frequency at the TS, is also given for each system.

**Figure 4:**
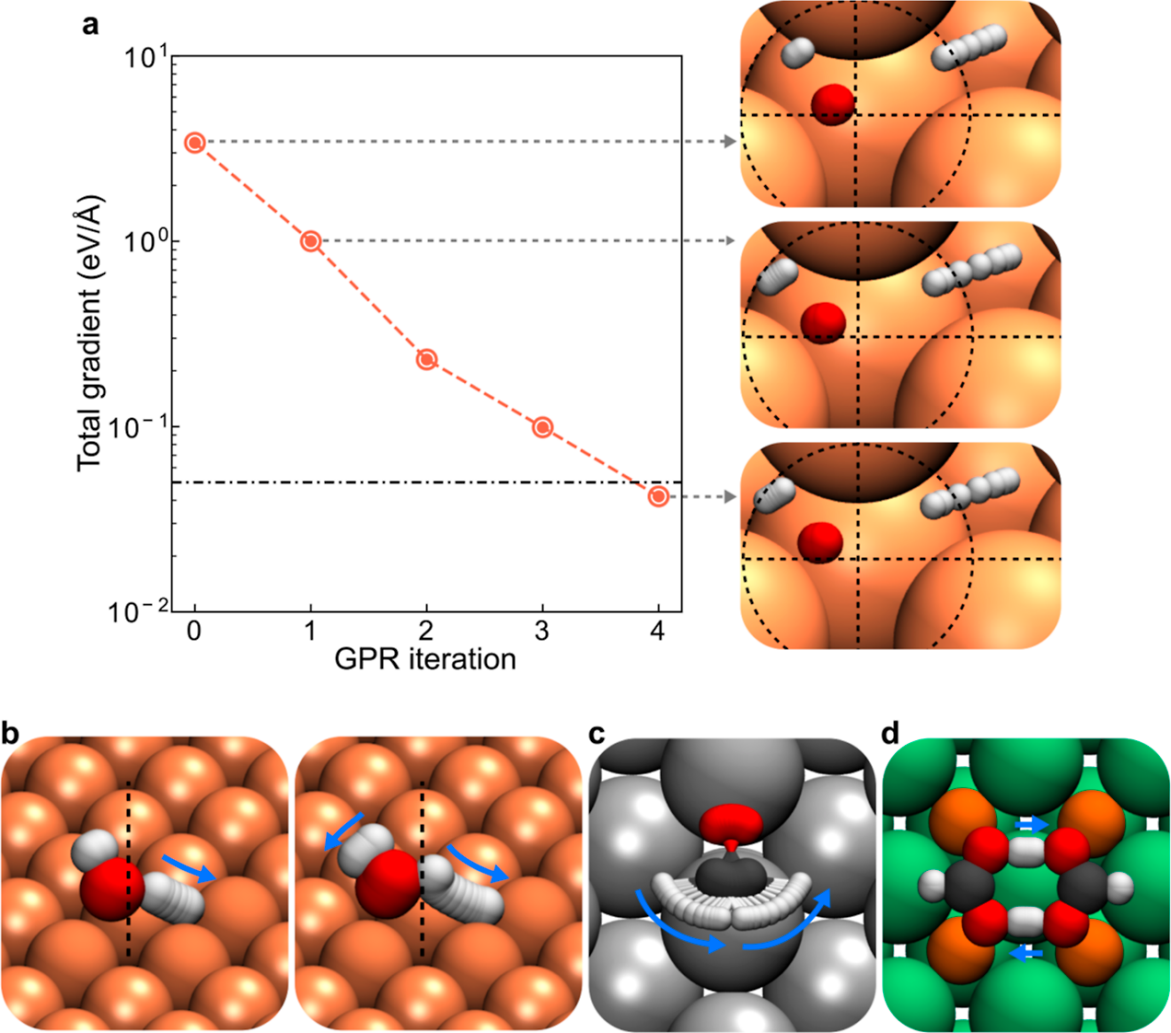
(a) Instanton geometries for dissociation on Cu(111) at 200 K at the 0th (initial guess), first, and final iteration during the GPR-assisted optimization. Norm of the ab initio total gradient during the optimization process is shown, and the dash-dotted line marks the convergence criteria. Optimized instanton geometries for H_2_O dissociation on Cu(111) (b) at 200 K (left) and 80 K (right), CH_2_O rotation on Ag(111) at 12 K (c), and DPT in FAD on NaCl(001) at 100 K (c). The flexible surface atoms in (b,c) are rendered in a different texture than the other substrate atoms. The dotted lines are guides for the eye, and the blue arrows show the movement of H atoms along the instanton.

Using the procedures described above, we performed ab initio instanton optimizations for the example systems at different temperatures in a “sequential cooling” manner. Note that the temperature gap between adjacent instanton optimizations is large, corresponding to a β increase of ∼50%. Encouragingly, all the GPR-assisted instanton optimizations successfully converged with ease, demonstrating that our GPR method is able to model all three types of processes: dissociation on the surface, rotation on the surface involving heavy-atom tunneling, and proton transfer between adsorbates. Examples of the optimized instantons are shown in Figure 4. Noticeably, even when the instanton displays significant corner-cutting effects (Figure 4b, indicated by the change in the position of the two instantons), our GPR method still performs well. To gain a straightforward view of the GPR-assisted optimization process, we show in Figure 4a the geometry and total ab initio gradient after each GPR iteration. The total gradient decreases exponentially after each GPR iteration, which is a sign of efficient and stable optimization.

In contrast, we performed ab initio instanton optimizations for these systems using conventional methods. There are a few conventional optimization algorithms that have been adapted for ab initio instanton optimization, belonging to two categories: mode-following methods (i.e., dimer methods)^48,49^ and Hessian-based methods (i.e., quasi-Newton methods).^26,47,50^ The performance of these instanton optimization algorithms have been discussed in detail in previous works.^46,47^ In short, Hessian-based methods are more stable and converge faster than mode-following methods but require calculation of the Hessians for the starting configuration, as they tend to fail for instanton optimization without a good estimate of the initial Hessian. This means that for systems with a relatively small number of flexible atoms, Hessian-based methods are overall more efficient, while for systems with many flexible atoms, the mode-following methods can be advantageous. Therefore, we use the quasi-Newton method described in ref (47) as the conventional instanton optimization method.

A full comparison of the convergence of ab initio instanton optimization with GPR and with the conventional method for all the 8 instanton optimizations is presented in Table 2. The results show that in all the cases tested, our GPR method clearly outperforms the conventional method for instanton optimization in surface reactions. With our GPR method, all these instantons appear to be easy to optimize, while for the conventional method, the opposite is true, as the majority of the optimizations takes many iterations. In several cases, the conventional optimization method becomes unstable after a number of iterations and requires a restart from a selected intermediate configuration (as well as recomputing the initial Hessian) in order to converge the optimization. This means that the actual number of ab initio energy and force evaluations on the instanton in these cases is even larger than the n_iter_ given in the table. Overall, one can see that our GPR method makes difficult optimizations feasible by reducing the number of ab initio calculations needed to converge the optimization by a factor of 5 or more.

**Table 2:** Comparison of the Convergence Speed (i.e., the Number of Iterations *n*_iter_) and Computational Cost between ab initio Instanton Optimization with GPR Assistance and That with Conventional Quasi-Newton Algorithm^a^

			GPR-assisted		conventional	
system	T (K)	N beads	*n*_iter_	*n*_hess_	*n*_iter_	*n*_hess_
H_2_O–Cu(111)	200	14	4	2	18	7
	130	30	4	5	15*	22
	80	50	6	5	18*	40
CH_2_O–Ag(110)	18	14	2	2	19*	14
	12	30	1	5	29*	22
	8	50	1	5	27	15
FAD–NaCl(001)	150	14	2	2	7	7
	100	30	1	5	not converged	

a *n*_hess_ is the number of ab initio Hessian evaluations performed. The superscript “*” means that the ab initio optimization required restarting from a selected intermediate geometry in the optimization process, and re-computing the Hessian on this geometry. In this case, the number of iterations actually performed is larger than *n*_iter_.

**Figure 5:**
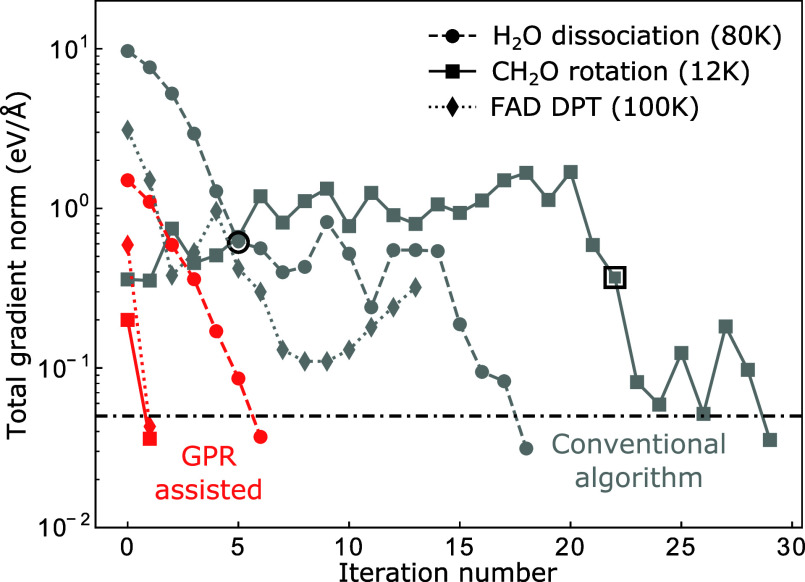
Norm of the total gradient at each iteration for selected ab initio instanton optimization with GPR assistance (red) and with the conventional algorithm (gray). The circled points mark the restart points of the ab initio instanton optimization.

To understand why GPR drastically outperforms the conventional method, we compare the change in the total gradient during the entire optimization process for the two methods (Figure 5). In the conventional instanton optimization for H_2_O dissociation and FAD DPT, during the first few iterations, the total gradient on the instanton decreases quite rapidly. However, afterward, the approximate Hessian becomes poor due to accumulation of errors from the updates. As a result, the optimization fails to further minimize the total gradient and a restart with a re-evaluation of the instanton Hessian (which is computationally demanding) is needed. At the core of this issue is that conventional methods barely utilize the ab initio data computed in previous iterations, wasting a lot of useful information and resulting in taking misguided steps. GPR exploits the data from previous iterations in-depth, learning information about the shape of the PES that provides guidance for the next optimization iteration, which can greatly accelerate the convergence without resorting to performing expensive Hessian calculations. A second advantage of GPR optimization is that it allows relatively major changes in the geometry after one iteration, such as in the first GPR iteration, as shown in Figure 4a, without encountering stability issues. Whereas with conventional optimization methods, taking small optimization steps means slow convergence, yet taking large steps can lead to instabilities. Thanks to the low computational cost of GPR PES compared to ab initio calculations, one can fully explore the GPR PES before needing to perform another expensive ab initio calculation.

**Figure 6:**
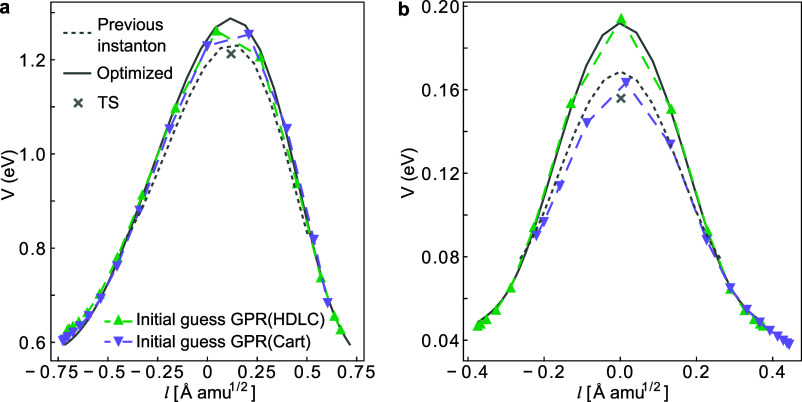
Comparison of the initial instanton guess predicted by HDLC-GPR PES and that predicted by Cartesian GPR PES for (a) H_2_O dissociation on Cu(111) at 130 K and (b) DPT in FAD on NaCl(001) at 100 K. The ring-polymer beads are plotted as a function of their potential energy and the path length *l*. For reference, the previous instanton (gray dotted line) from which the GPR PESs are trained and the final optimized instanton (gray solid line) are shown.

The in-depth learning power of our GPR method gives it a third key advantage over conventional methods: the ability to generate a good initial guess that is very close to the optimized instanton, using only data accumulated in the previous instanton optimization. This is indicated in Figure 5, where the GPR initial guesses have a significantly smaller ab initio gradient compared to the conventionally used initial guesses, which is another important reason why GPR-assisted optimization converged very fast. Here, we demonstrate that the in-depth learning is achieved by the use of the HDLC descriptor, while using Cartesian descriptor produces much worse results. We found that the HDLC-GPR initial guesses are almost identical to the optimized instanton (green vs gray lines in Figure 6), even when significant “corner-cutting effects” exist in the system. Corner cutting refers to when the optimal tunneling pathway (instanton) deviates from the MEP. Typically, corner cutting is more pronounced in the deep tunneling regime at low temperatures, while as the temperature increases, corner cutting becomes less significant and the instanton becomes closer to the MEP. This indicates that when strong corner cutting occurs, the instanton pathway at a lower temperature does not follow the instanton pathway at a higher temperature. It is particularly exciting that HDLC-GPR is able to capture corner-cutting effects quite well, given that machine-learning methods are generally not good at “extrapolation”.

In contrast, GPR with the Cartesian descriptor does not work well when corner-cutting effects exist in the system, failing to predict a good guess for the new instanton based on previous data. Generally speaking, when the low temperature instanton pathway does not follow the high temperature instanton pathway, it is difficult to correctly characterize the similarities and the differences between the two instantons from a Cartesian-coordinate perspective. Consequently, using data from one instanton may fail to predict the other. For FAD, the main difference between the instanton path and the MEP is that the in the MEP, the two monomers would first come closer together and then proton transfer (PT) occurs, whereas in the instanton path, at low temperatures, proton transfer occurs directly to avoid heavy-atom tunneling.^51^ At 150 K, the instanton pathway shows a thermally activated tunneling mechanism, where PT occurs when the two monomers are 3.64 Å apart, similar to the MEP. However, at 100 K, the instanton pathway features a deep tunneling mechanism, where PT occurs when the two monomers are 3.68 Å apart. With the Cartesian coordinate as the descriptor, it is evident that all coordinates differ between the two pathways, whereas in the case of HDLC, the intramolecular bond components in the descriptor remain the same. Therefore, HDLC can describe this system better than Cartesian coordinates. For H_2_O dissociation on Cu, the corner-cutting effect is less pronounced, hence the performance of Cartesian-GPR is not as poor. By examining the geometries, we find that HDLC-GPR predicts an initial guess that is closer to the true instanton, while the Cartesian-GPR initial guess basically followed the previous instanton, only extending the path length.

In general, the more “molecule-like” the surface reaction is, the more advantageous the HDLC descriptor is over Cartesians. On the other hand, we would not expect HDLC to outperform Cartesian coordinates for, e.g., H_2_ dissociation. At the very least, HDLC can serve as an alternative to the Cartesian descriptor in case the latter under-performs.

### 4.4 Selective Hessian Training

Finally, as a proof of concept, we demonstrate the performance of selective Hessian training on two systems: H_2_O dissociation and DPT in FAD. A straightforward way to select Hessian elements for surface systems is to divide them into three parts, i.e., the adsorbate part, the substrate part, and the adsorbate–substrate part, and select the desired parts. For the H_2_O dissociation instanton optimization at 130 K, the initial data set has 6 Hessians, which (as described previously) correspond to the classical TS, the end beads of the instanton at 200 K, and the first, middle (highest energy), and final beads of the initial instanton guess. We select only the adsorbate part for the first three Hessians and discard the substrate part of the Hessian on the middle bead. This reduces the size of the Hessian data by 46%. Using this reduced training set, we reperformed GPR-assisted instanton optimization and compared the performance to the previous results without selective Hessian training. Encouragingly, we find that the optimization also converges rapidly (Table 3), performing as well as GPR optimization with full Hessians.

**Table 3:** Convergence Speed (i.e., the Number of Iterations *n*_iter_) and Computational Cost of the Selective Hessian Training Approach^a^

system	*T* (K)	*N* beads	*n*_iter_	*n*_hess_	reduction
H_2_O–Cu(111)	130	30	4	5	46%
FAD–NaCl(001)	150	14	2	2	26%
(flexible substrate)	100	30	1	5	41%

a *n*_hess_ is the same as in Table 2. “Reduction” shows the percentage reduction in the size of the Hessian data in the training set with this approach.

For the FAD on NaCl(001) system, we reperformed the instanton optimization with 7 flexible surface atoms (closest to the adsorbate). This is very expensive to perform with conventional methods. With the GPR + selective Hessian approach, we are able to converge the instanton with minimal computational effort, i.e., 1 or 2 iterations and only a few Hessian calculations (Table 3). These systems tested pose no challenge for selective Hessian training, showing that this approach does not compromise performance; meanwhile, it reduces the size of the training set considerably, revealing the promising application potential of our GPR optimization approach in larger systems.

### 4.5 Converging Instanton Rates

In the previous section, we demonstrated instanton optimization with GPR, and next we discuss using GPR to obtain converged instanton rates. Equation 1 needs to be converged with respect to the number of beads (*N*), and at low temperatures, a large number of beads is often required. The “rigorous” approach is to only use GPR for instanton optimization and then perform ab initio calculations on all of the beads to obtain the rate. Despite the assistance of GPR optimization, computing instanton rates rigorously can still be computationally expensive, especially at low temperatures, due to the ab initio Hessian calculations on a large number of beads. Alternatively, one can use a more “approximate” approach, which is to train GPR using the data of the instanton with a small number of beads and compute instanton rates with a large number of beads on the GPR PES.^31^ We benchmark the approximate approach against the rigorous instanton rate for H_2_O dissociation on Cu(111) at 80 K as an example.

Using the rigorous approach described above, we obtained ab initio instanton rates at 80 K with 50 and 130 beads. Here, we explore the accuracy of instanton rates computed with the computationally inexpensive approximate approach, with respect to the training set size. The training data set contains the energy and gradient data for all the beads in the optimized instanton at this temperature with 50 beads (which has 25 geometries as the instanton folds on itself). Hessian data on selected geometries (ensuring that these geometries are roughly evenly spaced along the instanton path) are also added.

The results are compared in Table 4. We find that the accuracy of the approximate approach can depend on the number of Hessian data in the training set, as one can see, using 7 Hessians results in large errors of over 100%, whereas using 10 Hessians, the error decreases to ∼15%. The instanton rate computed on the GPR PES with 130 beads has an error of ∼20% compared to the benchmark, which is acceptable, as this is comparable to the error of instanton theory itself. In principle, adding more ab initio data to the training set can further reduce the error (to only ∼1% in this example), but might not be necessary. One thing to note that for this reaction, at 80 K, using 50 beads results in a factor of ∼4 error in the rate and ∼70% error in the tunneling factor compared to the result with 130 beads, indicating that converging instanton rates may require many beads at low temperatures. GPR is a very efficient method for converging ab initio instanton rates, especially when many beads are required.

**Table 4:** Comparison of the Instanton Rate Computed on the GPR PES and the ab initio Instanton Rate for H_2_O Dissociation on Cu(111) at 80 K^a^

PES	*N*	*n*_hess_	*n*_ener,grad_	*S*/*ℏ*	*k*_inst_ (s^–1^)	tunneling factor	error (%)
DFT	50			116.032	5.11 × 10^–^^31^	5.47 × 10^22^	
GPR	50	7	25	116.030	3.03 × 10^–^^30^	3.25 × 10^23^	>100
GPR	50	10	25	116.030	4.33 × 10^–^^31^	4.64 × 10^22^	15.2
DFT	130			116.144	1.85 × 10^–^^30^	3.37 × 10^22^	
GPR	130	10	25	116.156	1.44 × 10^–^^30^	2.62 × 10^22^	22.2
GPR	130	10	90	116.157	1.86 × 10^–^^30^	3.40 × 10^22^	0.7

a The ab initio instantons were optimized with GPR assistance. The tunneling factor is defined as the ratio of the *N* bead instanton rate and the Eyring TST rate in the *N* bead limit.

The good performance of the “approximate” approach clearly implies that the computational cost of instanton rate calculations can be significantly reduced with GPR assistance without sacrificing much accuracy, making the cost comparable to that of a classical TST rate calculation. If we assume that the Hessian calculation is the bottleneck, the TST calculation requires 2 to 3 Hessians. With the help of GPR, an instanton calculation takes 3–8 Hessians for the optimization and ∼10 Hessians (depending on the temperature, the higher the fewer) for obtaining the rate. In contrast, instanton calculation carried out conventionally would be ∼2 orders of magnitude more expensive than TST, e.g., for the system demonstrated, it would need over 100 Hessian calculations. This means that performing a GPR instanton calculation is less than an order of magnitude more expensive than TST, while being orders of magnitude closer to the correct result for reactions where quantum tunneling plays an important role.

## 5 Conclusions

We have proposed a robust method for GPR-assisted geometry optimization with general descriptors and an improved descriptor (HDLC) over Cartesians for surface systems. The improved GPR method has several advantages over the previous method, including the fact that it no longer performs transformations of physical observables, which avoids associated numerical issues. The HDLC descriptor provides a more intrinsic and faithful description of surface systems, thus improving the performance of GPR. Ab initio instanton optimizations for surface reactions can be made efficient using HDLC-GPR to fit the PES locally around the tunneling path even for cases where significant corner-cutting occurs. This is demonstrated using three example systems representing different type of surface reactions and processes. GPR-assisted instanton optimization can obtain converged instantons with just a few iterations, truly a significant speed up from conventional optimization methods. This method brings down the cost for performing an instanton calculation to within an order of magnitude of the cost of a classical TST calculation, meaning that if TST is affordable, there is no reason not to perform an instanton calculation if there might be tunneling effects. We attribute the good performance to the HDLC-GPR method achieving in-depth learning of the data generated in the optimization process.

We postulate that the method proposed in this work has extensive application potential beyond what we have explored here. The HDLC descriptor is obviously applicable beyond surface systems, e.g., it can be used to describe processes in condensed-phase systems, offering a better alternative to the Cartesian descriptor. Our improved GPR framework for general coordinates can also alleviate some of the issues that GPR optimization faces. Most noticeably, selective Hessian training under the improved framework can allow GPR optimization to be applied to larger systems, and we have demonstrated that it reduces the cost while preserving good performance. We expect that GPR-based optimization schemes will replace conventional methods for the more difficult optimization calculations in the future. We are optimistic that the developments in this work bring us toward easy computation of reaction and tunneling pathways.
